# Targeting p70S6K1 Inhibits Glycated Albumin-Induced Triple-Negative Breast Cancer Cell Invasion and Overexpression of Galectin-3, a Potential Prognostic Marker in Diabetic Patients with Invasive Breast Cancer

**DOI:** 10.3390/biomedicines13030612

**Published:** 2025-03-03

**Authors:** Fatimah Alanazi, Abdulmonem A. Alsaleh, Mariam K. Alamoudi, Abdulrahman Alasiri, Amanda Haymond, Sabine Matou-Nasri

**Affiliations:** 1 Blood and Cancer Research Department, King Abdullah International Medical Research Center (KAIMRC), King Saud bin Abdulaziz University for Health Sciences (KSAU-HS), Ministry of National Guard-Health Affairs (MNG-HA), Riyadh 11481, Saudi Arabia; falanaz4@gmu.edu (F.A.); alsalehabd@kaimrc.edu.sa (A.A.A.); 2 Center for Applied Proteomics and Molecular Medicine, School of Systems Biology, George Mason University, Fairfax, VA 22030, USA; ahaymond@gmu.edu; 3 Department of Pharmacology, College of Pharmacy, Prince Sattam Bin Abdulaziz University, Al-Kharj 11942, Saudi Arabia; m.alamoudi@psau.edu.sa; 4 Department of Artificial Intelligence and Bioinformatics, KAIMRC, KSAU-HS, MNG-HA, Riyadh 11481, Saudi Arabia; asiriabdu@kaimrc.edu.sa

**Keywords:** ribosomal p70S6K1, glycated albumin, advanced glycation end products, triple-negative breast cancer, type 2 diabetes mellitus, PF-4708671, galectin-3

## Abstract

**Background:** There is an urgent need to identify new biomarkers for early diagnosis and development of therapeutic strategies for diabetes mellitus (DM) patients who have invasive breast cancer (BC). We previously reported the increased activated form of 70 kDa ribosomal protein S6 kinase 1 (phospho-p70S6K1) in a triple-negative BC (TNBC) cell line MDA-MB-231 exposed to glycated albumin (GA) and in invasive ductal carcinoma tissues from T2DM patients, compared to untreated cells and their non-diabetic counterparts, respectively. **Objective:** We aimed to explore the function of p70S6K1 in GA-promoted TNBC progression. **Methods:** By employing small interference (si)RNA technology or blocking its kinase activity using its specific pharmacological inhibitor, we monitored cell invasion using Transwell^®^ inserts and the expression levels of activated signaling proteins and cancer-related proteins using Western blot. **Results:** In silico analysis revealed that high mRNA levels of p70S6K1 were associated with an unfavorable prognosis and progression to advanced stages of TNBC in DM patients. The downregulation/blockade of p70S6K1 inhibited GA-promoted MDA-MB-231 cell invasion and the phosphorylation of protein S6 and ERK1/2, the p70S6K1 downstream effector, and the key oncogenic signaling protein, respectively. The suppression of the expression of GA-upregulated cancer proteins, including enolase-2, capping protein CapG, galectin-3, and cathepsin D, was observed after p70S6K1 downregulation/blockade. Further in silico validation analyses revealed increased gene expression of galectin-3 in DM TNBC patients, resulting in poor overall survival and disease-free survival. **Conclusions:** Targeting p70S6K1 may present a valuable therapeutic strategy, while galectin-3 could serve as a potential prognostic biomarker for invasive BC progression in DM patients.

## 1. Introduction

Diabetes mellitus (DM) is recognized as the most prevalent chronic metabolic disease globally. More than half a billion people are affected by DM worldwide, and this number is expected to increase in the coming decades [1]. DM is mainly characterized by uncontrolled high glucose levels that result from insufficient insulin production by pancreatic β-cells, affecting the transport of glucose to the required cells [2]. Several studies have reported that patients with type 2 DM (T2DM), the main type of DM, are at a higher risk of developing various diseases, including hypertension, cardiovascular diseases, kidney failure, as well as different types of cancer, including liver cancer, colorectal cancer, and breast cancer (BC) [3]. BC is the most prevalent cancer among women worldwide and ranks as the second leading cause of cancer-related deaths in the United States. It is a heterogeneous disease that is categorized into four primary molecular subtypes: luminal A, luminal B, human epidermal growth factor receptor (HER)-2 enriched, and basal-like/triple-negative [4,5]. Studies conducted on retrospective cohorts have indicated that inadequate glycemic control is a risk factor for triple-negative BC (TNBC) progression in T2DM patients [6]. TNBC is a typical subtype of BC with the absence of the progesterone receptor (PR), estrogen receptor (ER), and HER-2 overexpression. TNBC is the most aggressive subtype among all forms of BC with a high recurrence rate, strong invasiveness, and poor prognosis due to the lack of targeted therapy because of the absence of hormone receptor expression [7].

Persistently elevated glucose levels, defined as hyperglycemia in diabetic patients, result in the glycation process, which is referred to as a Maillard reaction [8]. It is a non-enzymatic reaction that occurs between the free amino groups of proteins, lipids, and nucleic acids and the carbonyl groups of reducing sugars. This reaction generates advanced glycation end products (AGEs), which are involved in many diabetic complications [8]. AGEs induce multiple changes in the structure of glycated proteins, which alter the protein’s function and cause deleterious effects: the generation of free radicals, platelet activation and aggregation, and immune system dysregulation [8]. Albumin is the plasma protein most frequently exposed to the glycation reaction, which reduces its functionality of transporting lipids and steroid hormones [8]. Glycated albumin bound to the main receptor for AGEs (RAGE) promotes free-radical activation, genetic mutation, inflammation, and tumorigenesis, thereby leading to tumor progression. An in vitro study reported the impact of methylglyoxal-derived glycated albumin (i.e., AGEs) on MDA-MB-231 (a TNBC model) cell invasion, migration, and proliferation compared to non-treated cells [9]. This in vitro investigation revealed that ribosomal protein S6 kinase beta-1 (p70S6K1) is overphosphorylated in glycated albumin-treated MDA-MB-231 cells. Ribosomal p70S6K1 is a downstream target of the oncogenic signaling pathway phosphatidylinositol 3-kinase (PI3K)/Akt/mammalian target of rapamycin (mTOR) through several phosphorylation cascades, which is mainly stimulated by the insulin receptor [8]. Ribosomal p70S6K1 plays crucial roles in several cell processes such as cell proliferation, apoptosis suppression, drug resistance, and cell invasion [10].

Numerous epidemiological investigations have indicated that T2DM women face a 20–30% increased risk of TNBC onset, development, and progression when compared to women without DM [11,12,13,14]. Microarray analysis proved that TNBC is the most commonly associated BC subtype with T2DM women among other subtypes [15]. The hyperglycemic tumor microenvironment, including oxidative stress, glucose transporter alterations, and immune cell exhaustion, promotes the initiation and development of BC, particularly TNBC [16,17,18]. Moreover, postmenopausal women with T2DM are more likely to be diagnosed with an advanced stage of TNBC, compared with non-DM cases, and they are at a very high risk of invasive BC [19]. It is also noteworthy that during chemotherapy, cancer patients are at a high risk of developing insulin resistance and subsequently being diagnosed with T2DM [20]. Additionally, a prospective sister study found that the prolonged use of metformin was inversely associated with the risk of ER-positive BC but was associated with an increased risk of TNBC in T2DM patients [21]. Immunohistochemical staining recently revealed elevated p70S6K1 phosphorylation in invasive ductal carcinoma (IDC) tissues extracted from postmenopausal women with T2DM, in contrast to their non-DM counterparts [14], suggesting p70S6K1 overphosphorylation may serve as a therapeutic target and potential biomarker for invasive BC in T2DM patients. In addition, interlinking metabolic association between T2DM and cancer has been widely reported, suggesting the metabolic pathway mTOR/p70S6K1 and mTOR inhibitors, prescribed to overcome hormone receptor-positive BC resistance to hormone therapy, as emerging therapeutic targets against metastatic BC [22,23,24]. Therefore, the central role of p70S6K1 in TNBC invasion upon glycated albumin treatment was investigated after downregulating p70S6K1 gene expression using small interfering (si)RNA and blocking its activity using a pharmacological p70S6K1 inhibitor named PF-4708671.

## 2. Materials and Methods

### 2.1. Bioinformatics

Molecular Taxonomy of Breast Cancer International Consortium (METABRIC) data from cBioPortal (https://www.cbioportal.org/; accessed on 21 October 2024) [25,26,27,28,29] were utilized for the overall survival (OS) study of p70S6K1. In the cBioPortal under “breast” section, a dataset labeled “breast invasive carcinoma” (TCGA, Firehose Legacy) was downloaded (accessed on 21 October 2024) [30]. Patients were divided into low- and high-expression groups based on the median values of the investigated genes. mRNA expression z-scores relative to all samples (log microarray) were used for analysis. TNBC patients’ data were selected based on a negative immunohistochemical status of ER, PR, and HER2. The patients’ data were downloaded from the survival section, accordingly. The MammOnc-DB (SCAN-B and Creighton breast tumor compendium) database (http://resource.path.uab.edu/MammOnc-Home.html; accessed on 21 October 2024) was employed to assess p70S6K1 mRNA expression in relation to lymph node status and a Prediction Analysis of Microarray (PAM) of 50 subtypes [31]. Data were acquired from the Swedish Cancerome Analysis Network-Breast (SCAN-B) and the Creighton breast tumor compendium datasets, respectively [32,33]. METABRIC and MammOnc-DB datasets were selected because they include curated expression data for a large number of BC patients (*n* = 2509, 9206 and 961, respectively).

Data from the study of Tamez-Peña and colleagues were retrieved from the GEO data repository (GSE75678; accessed on 21 October 2024). The processed signal intensity data were used for pairwise comparison between DM and non-DM TNBC patients [34].

### 2.2. Generation of Glycated Albumin

Methylglyoxal-derived bovine serum albumin-advanced glycation end products (MG-BSA-AGEs), hereafter called glycated albumin (GA), were prepared as previously described [9]. Briefly, BSA fraction V was incubated with methylglyoxyl for 72 h under physiological conditions (pH 7.4, 37 °C) to prepare MG-BSA-AGES, which were subsequently dialyzed into distilled water to remove unbound sugars prior to use.

### 2.3. MDA-MB-231 Cell Culture and Treatment

The MDA-MB-231 TNBC cell line (#HTB-26™), sourced from the American Type Culture Collection (Manassas, VA, USA), was maintained in a complete medium containing Dulbecco’s Modified Eagle’s medium (DMEM) enriched with 10% heat-inactivated fetal bovine serum (FBS), 2 mM of glutamine and 1% antibiotics (100 μg/mL of streptomycin, 100 IU/mL of penicillin) at 37 °C in a humidified atmosphere/5% CO_2_ incubator. The complete medium was refreshed every 2–3 days, and the confluent cells were subcultured using the enzymatic solution TrypLE™ Express (Thermo Fisher Scientific, Waltham, MA, USA). For all in vitro experiments, cells were utilized at passages 5–10.

Prior to treatment, cells were exposed to the specific pharmacological p70S6K1 inhibitor PF-4708671 (#sc-361288, Santa Cruz Biotechnology, Heidelberg, Germany) at a concentration of 10 μM (the optimal concentration determined from preliminary studies) along with 0.04% dimethyl sulfoxide (DMSO, the solvent for the reconstitution of PF-4708671) for an incubation of 1 h. Throughout the experiment, cells were exposed to 100 μg/mL of GA.

### 2.4. p70S6K1 Knockdown Using siRNA Technology

MDA-MB-231 cells were reverse transfected with a designed siRNA targeting human p70S6K1 (#sc-36165 composed of two siRNA duplexes #sc-36165A sense: 5′-CAAGGUCAUGUGAAACUAAdTdT-3′, antisense: 5′-UUAGUUUCACAUGACCUUGdTdT-3′; #sc-36165B sense: 5′-GAGAGUCAAUGUCAUUACAdTdT-3′, antisense: 5′-UGUAAUGACAUUGACUCUCdTdT-3′ Santa Cruz Biotechnology) complexed with the RNAiMax transfection reagent (Invitrogen, Carlsbad, CA, USA). Briefly, 100 nM of p70S6K1 siRNA and scrambled negative control (NC) siRNA (#sc-37007, Santa Cruz Biotechnology) were transfected into 70–80% confluent MDA-MB-231 cells (1.5 × 10^5^ cells/well) and grown in a 24-well plate in basal medium free of serum and antibiotics. Cells were incubated for 2 h before adding 10% FBS with 1% antibiotics then cultured for an additional 48 h of incubation. Transfected and untransfected cells were utilized for downstream applications or protein extraction to verify p70S6K1 downregulation using Western blot analysis. Each condition was carried out at least twice, and each experiment was performed independently three times.

### 2.5. Cell Lysate Preparation and Western Blot Technology

As previously described [35], cell lysates were prepared, proteins were separated using 12% sodium dodecyl sulfate–polyacrylamide gel electrophoresis, and the separated proteins were transferred to polyvinylidene difluoride membranes. After that, the membranes were incubated with primary antibodies that had been diluted in Odyssey^®^ intercept buffer for an entire night at 4 °C on a rotating shaker. The primary antibodies, including mouse monoclonal antibodies against phospho-p70S6K1 [E-5] (p-p70S6K1, Thr421/Ser424, #sc-377529, and 1:1000 dilution), p70S6K1 [H-9] (#sc-8418, 1:1000 dilution), phospho-ribosomal protein S6 (p-S6, 50.Ser235/236, #sc-293144, and 1:1000 dilution), ribosomal protein S6 [C8] (#sc-74459, 1:1000), phospho-ERK1/2 [E-4] (p-ERK1/2, Tyr204 of ERK1, #sc-7383, and 1:1000 dilution), ERK1/2 [G-8] (#sc-271269, 1:1000 dilution), cathepsin D [D-7] (#sc-377299, dilution 1:500), CapG [H-9] (#sc-166428, 1:500), as well as rabbit monoclonal antibodies against enolase-2 [NSE-P1] (#sc-21738, 1:500) and galectin-3 [B2C10] (#sc-32790, dilution 1:500), were provided by Santa Cruz Biotechnology. Mouse monoclonal antibody against GAPDH [6C5] (#ab8245, 1:2000 dilution) was purchased from Abcam (Cambridge, UK). Infrared fluorescent IRDye^®^ 800RD (green)-conjugated goat anti-mouse and IRDye^®^ 700RD (red)-conjugated goat anti-rabbit secondary antibodies (LI-COR Biosciences, Lincoln, NE, USA) that were diluted (1:5000 dilution) in Odyssey^®^ blocking buffers and incubated for 1 h at room temperature with constant agitation were used to detect primary antibodies. Blots were washed then scanned, examined, and quantified using the LI-COR Odyssey CLx Scanner (LI-COR Biosciences, Lincoln, NE, USA) and ImageJ software version 1.53e (https://imagej.net/ij/index.html, accessed on 2 March 2023).

### 2.6. Cell Invasion Assay

The Boyden chamber system, utilizing Transwell^®^ inserts (Nunc™, Thermo Fisher Scientific, Waltham, MA, USA) placed in a 24-well plate, was employed to evaluate the invasion of MDA-MB-231 cells as previously described [9]. Untransfected and transfected MDA-MB-231 cells (10^4^/100 μL) with either NC siRNA or p70S6K1 siRNA, untreated, and DMSO- and PF-4708671-pretreated cells were seeded onto a porous membrane layered with Corning^™^ growth factor-reduced Matrigel^®^ (#356230, Thermo Fisher Scientific), a reconstituted basement membrane, in serum-poor medium (SPM) situated in the upper chamber of the insert, while the lower chamber (the well) contained SPM with or without GA. Each condition was replicated in duplicate, and the entire experiment was independently conducted three times. Following a 24 h incubation period, the cells that had not crossed the porous membrane were eliminated using cotton swabs soaked in PBS while the cells that had invaded were fixed, stained, and counted according to the previously established methodology [35].

### 2.7. Oncology Protein Array

The detection of cancer-related proteins, including enzymes, signaling proteins, and extracellular matrix proteins, was carried out using the Proteome Profiler Human Oncology protein array kit (#ARY026, Life Technology, Thermo Fisher Scientific). Briefly, both untransfected MDA-MB-231 cells (1.5 × 10^6^/mL) and cells transfected with NC siRNA or p70S6K1 siRNA cells were incubated with or without GA for 48 h. Following incubation, cells were subjected to centrifugation at 200× *g* for 5 min. The resulting cell pellet was lysed utilizing the lysis buffer supplied by the manufacturer. All procedures, from protein extraction to detection using enhanced chemiluminescence (ECL), were carried out according to the instructions provided by the manufacturer. The proteins appeared as dark spots on a white background, and their expression levels were quantified using densitometry with Image J software v1.53e.

### 2.8. Statistical Analysis

Experimental results are shown as mean ± standard deviation (SD) from three separate assays. One-way analysis of variance (ANOVA) was used for data comparison, and Tukey’s test was applied for the comparison of the groups. OS and disease-free survival (DFS) analyses were conducted using the log-rank test. Kaplan–Meier plots were produced using GraphPad Prism 10.0, RStudio “survival-version 3.7.0”, and “survminer-version 0.4.9” packages. Each *p* value of less than 0.05 was considered statistically significant.

## 3. Results

### 3.1. Prognostic Impact of p70S6K1 Expression Levels on BC Patient Overall Survival

**Figure 1:**
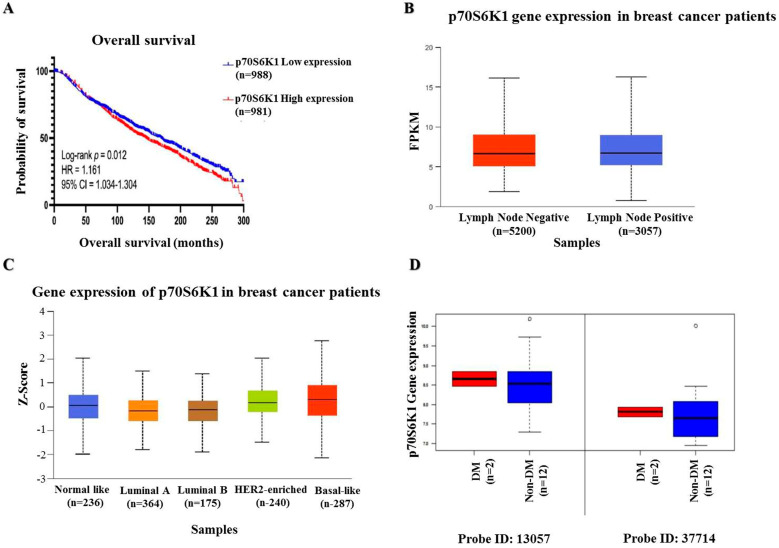
**Bio-statistical analysis.** (**A**) Kaplan–Meier plot of p70S6K1 overall survival (OS) in patients with invasive BC showing poor-survival patients with high p70S6K1 gene expression levels. Data were collected from the METABRIC dataset within cBioPortal. (**B**) The mRNA expression of p70S6K1 based on lymph node status reveals high p70S6K1 gene expression in patients with lymph node metastases (lymph node positive). Data were obtained from the SCAN-B dataset within MammOnc-DB. FPKM: Fragments per kilobase of transcript per million mapped reads. (**C**) The mRNA expression of p70S6K1 according to PAM50 subtypes indicates a higher gene expression of p70S6K1 among the basal-like BC subtype compared to other subtypes. Data were derived from the Creighton breast tumor compendium within MammOnc-DB. (**D**) The mRNA expression of p70S6K1 among DM and non-DM TNBC patients shows increased p70S6K1 gene expression among diabetic TNBC patients (data point outside the error bars is an outlier). Data were obtained from the study of Tamez-Peña et al. [34]. The log-rank test was employed for OS analysis. The Kaplan–Meier plot was created using GraphPad Prism 10.0. The middle line of the boxplots represents the median value; the error bars represent the maximum and minimum values.

To assess the impact of p70S6K1 expression on invasive BC patient survival, data were extracted from the METABRIC dataset in cBioPortal. Patients were categorized into low and high p70S6K1 expression groups according to the median values. Figure 1A illustrates that survival analysis indicated that patients with low mRNA expression levels of p70S6K1 (*n* = 988) exhibited significantly prolonged OS, with a median survival of 169.2 months, in contrast to those with high expression (*n* = 981), who had a median survival of 145.4 months (HR = 1.161; 95% CI = 1.034–1.304; *p* = 0.012). Further investigation was conducted utilizing the MammOnc-DB database. The SCAN-B cohort was divided into two groups depending on lymph node status: lymph node-negative BC patients (*n* = 5200) and lymph node-positive BC patients (*n* = 3057). Figure 1B shows that BC patients who were lymph node positive had significantly elevated p70S6K1 mRNA expression (*p* = 7.758 × 10^−4^). Analyzing the Creighton breast tumor compendium according to the PAM50 subtyping, as observed in Figure 1C, we found that the p70S6K1 mRNA expression level was significantly higher in basal-like (*n* = 287) subtypes compared with normal-like (*n* = 236), luminal A (*n* = 364), and HER2-enriched (*n* = 240) subtypes (*p* = 4.850 × 10^−4^, 4.401 × 10^−4^, and 9.365 × 10^−4^, respectively). Data from the study of Tamez-Peña et al. included 54 BC patients, 14 of which were diagnosed with TNBC [34]. Two of the TNBC patients were diabetic (DM) and twelve were non-diabetic (non-DM). Analysis of p70S6K1 mRNA expression (two probes: 13057 and 37714) showed higher expression levels among diabetic TNBC patients than their non-diabetic counterparts (Figure 1D).

### 3.2. Knockdown of p70S6K1 Inhibits GA-Induced MDA-MB-231 Cell Invasion

**Figure 2:**
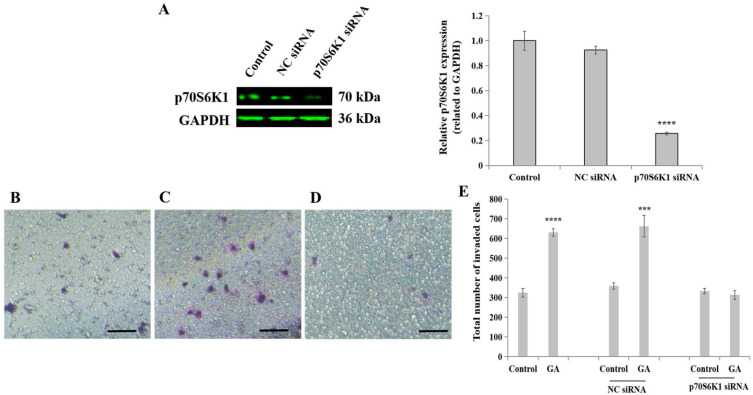
**Impact of p70S6K1 downregulation using siRNA technology on glycated albumin (GA)-stimulated MDA-MB-231 cell invasion.** (**A**) Representative Western blot showing p70S6K1 downregulation after 48 h of transfection using p70S6K1 siRNA, compared with the basal level of p70S6K1 detected in untransfected and negative control (NC) siRNA-transfected MDA-MB-231 cells. The bar graph shows the relative expression of p70S6K1 expressed in pixel density and related to GAPDH, the loading control. (**B**–**E**) Representative photomicrographs showing the stained and invaded untreated MDA-MB-231 cells (**B**), NC siRNA-transfected cells treated with GA (**C**), and p70S6K1 siRNA-transfected cells treated with GA (**D**) using the Boyden chamber system. Scale bar: 100 μm. (**E**) The bar graph shows the number of invaded MDA-MB-231 cells, NC siRNA, and p70S6K1 siRNA-transfected cells treated with or without GA, compared to the untreated cells, the control. The results are presented as the mean ± SD of three independent experiments. (***) and (****) signify a statistically significant difference (*p* < 0.001 and *p* < 0.0001) compared with the control.

To reveal the role of the ribosomal protein p70S6K1 in invasive BC progression in (T2)DM patients, particularly involving cell invasion processes, siRNA transfection technology was first applied to downregulate p70S6K1 expression in highly invasive TNBC cell line MDA-MB-231. The concomitant downregulation of p70S6K1 was obtained after a 2-day transfection of MDA-MB-231 cells with p70S6K1 siRNA. Using Western blot analysis, a 75–80% decrease in the p70S6K1 expression level was observed in p70S6K1 siRNA-transfected MDA-MB-231 cells compared to the basal level of p70S6K1 detected in untransfected cells (control) and in NC siRNA-transfected cells (Figure 2A).

In the presence or absence of GA, the biological impact of p70S6K1 on cell invasion was evaluated using insert systems containing porous membranes coated with growth factor-reduced Matrigel^®^ (Thermo Fisher Scientific). The addition of GA significantly increased the number of invaded cells by 1.94-fold (*p* = 0.000053, Figure 2E) in untransfected cells and by 2.03-fold (*p* = 0.00059, Figure 2E) in NC siRNA-transfected cells (Figure 2B), as compared with untreated cells (Figure 2A,E) and untreated NC siRNA-transfected cells (Figure 2E). The loss of the stimulatory effect of GA on TNBC cell invasion was observed in p70S6K1 siRNA-transfected cells (Figure 2D), resulting in a similar number of invaded cells as untreated untransfected cells, even after exposure of the cells to GA (Figure 2E).

### 3.3. Knockdown of p70S6K1 Inhibits GA-Induced p70S6K1 and ERK1/2 Phosphorylation in MDA-MB-231 Cells

**Figure 3:**
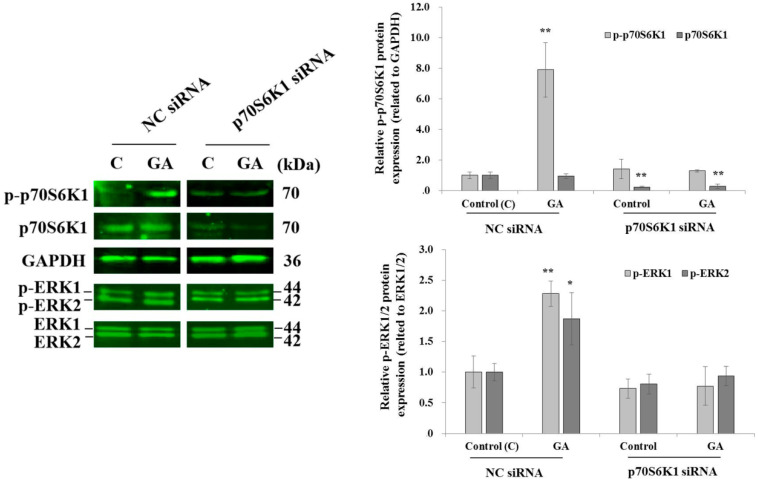
**Inhibition of glycated albumin (GA)-induced p70S6K1 and ERK1/2 phosphorylation in MDA-MB-231 cells after p70S6K1 downregulation.** Representative Western blots showing the impact of p70S6K1 downregulation on GA-induced p70S6K1 and ERK1/2 phosphorylation compared with the negative control (NC) siRNA-transfected MDA-MB-231 cells. The results are presented as the mean ± SD of three independent experiments. (*) and (**) signify a statistically significant difference (*p* < 0.05 and *p* < 0.01) compared with the control.

**Figure 4:**
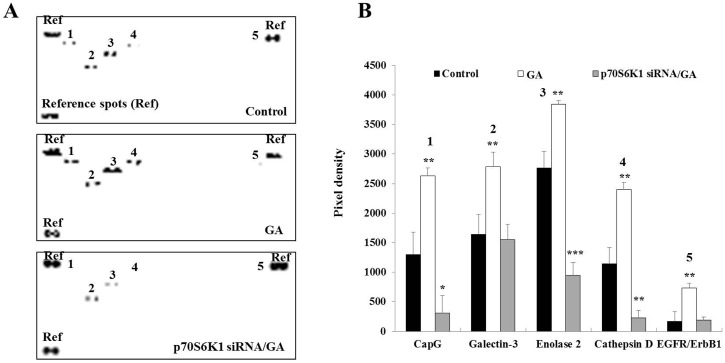
**Decrease in glycated albumin (GA)-induced cancer-related protein expression in MDA-MB-231 cells after p70S6K1 downregulation.** (**A**) Representative oncology array membranes of cancer-related proteins detected in untreated MDA-MB-231 cells, cells treated with GA, and negative control (NC) siRNA- and p70S6K1 siRNA-transfected MDA-MB-231 cells treated with GA, after 48 h of incubation. (**B**) The bar graphs show the relative expression of the relevant cancer-related proteins expressed in pixel density based on the similar brightness of spots considered references. The results are presented as the mean ± SD of three independent experiments. (*), (**), and (***) signify a statistically significant difference (*p* < 0.05, *p* < 0.01, and *p* < 0.001) compared with the control.

After 10 min of MDA-MB-231 cell exposure to GA, a significant increase in p70S6K1 phosphorylation was observed in cells transfected with NC siRNA, compared to the p70S6K1 phosphorylation level detected in untreated NC siRNA-transfected cells (Figure 3). No changes in the phosphorylation levels of p70S6K1 were observed in p70S6K1 siRNA-transfected cells, even after exposure to GA (Figure 4). Similar variations in the ERK1/2 phosphorylation level were observed, such as an increase in GA-induced ERK1/2 phosphorylation and a decrease in GA-induced ERK1/2 phosphorylation after p70S6K1 downregulation (Figure 3).

### 3.4. Knockdown of p70S6K1 Suppresses GA-Upregulated Cancer-Related Protein Expression in MDA-MB-231 Cells

Cancer-related proteins play a major role in cancer development and progression. To gain an overview of the expression of the main cancer-related proteins upregulated by GA, an oncology array was performed using whole cell lysates after a 2-day incubation of untransfected, NC siRNA-transfected, and p70S6K1 siRNA-transfected MDA-MB-231 cells in the presence or absence of GA. Compared to untreated cells, GA significantly increased the expression of CapG (2.02-fold, *p* = 0.0047), galectin-3 (1.69-fold, *p* = 0.0084), enolase-2 (1.38-fold, *p* = 0.0028), cathepsin D (2.09-fold, *p* = 0.0019), and EGFR/ErbB1 (4.26-fold, *p* = 0.054) (Figure 4). Similar changes in the expression of these proteins were observed in GA-treated NC siRNA-transfected cells. The impact of p70S6K1 downregulation resulted in the suppression of GA-increased galectin-3 and EGFR/ErbB1 expression, reaching a level similar to the basal expression level detected in untreated cells (Figure 4). Compared to the basal level of expression of CapG, enolase-2 and cathepsin D detected in untreated cells, a significant inhibition of GA-increased CapG (0.23-fold, *p* = 0.024), enolase-2 (0.34-fold, *p* = 0.00084), and cathepsin D (0.19-fold, *p* = 0.0064) expression levels was observed after p70S6K1 downregulation (Figure 4).

### 3.5. Blockade of p70S6K1 Pathway Using PF-4708671 Inhibits GA-Induced MDA-MB-231 Cell Invasion, Ribosomal Protein S6, ERK1/2 Phosphorylation, and Cancer-Related Protein Expression

**Figure 5:**
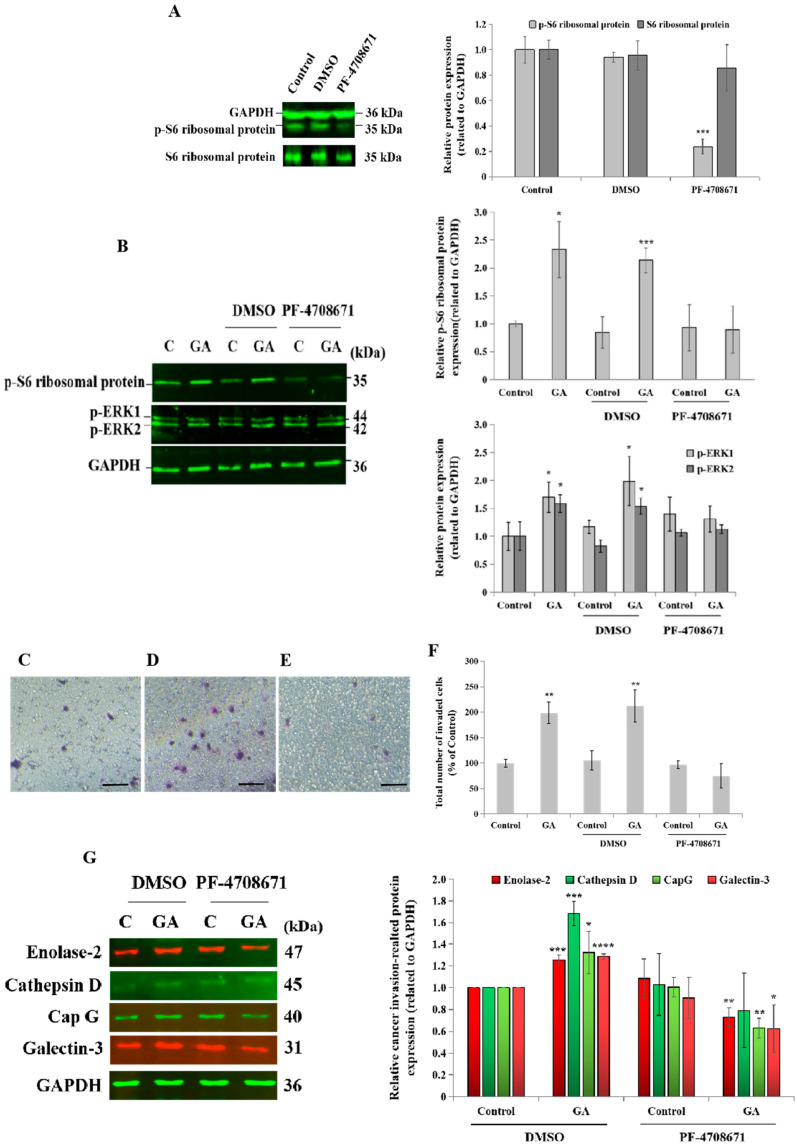
**Impact of p70S6K1 pathway blockade by PF-4708671 on glycated albumin (GA)-induced cell invasion, signaling pathways, and cancer-related protein expression in MDA-MB-231 cells.** (**A**) Representative Western blot showing the decrease in the S6 ribosomal protein phosphorylation level in MDA-MB-231 cells after 1 h of treatment with the pharmacological p70S6K1 inhibitor PF-4708671, compared with the S6 ribosomal protein phosphorylation level detected in untreated cells (control) and DMSO-treated cells. PF-4708671 did not affect the total S6 ribosomal protein expression. GAPDH was used as the loading control. (**B**) Representative Western blots showing the impact of the p70S6K1 blockade on GA-induced S6 ribosomal protein and ERK1/2 phosphorylation compared with the control and DMSO-pretreated MDA-MB-231 cells. (**C**–**F**) Representative photomicrographs showing the stained and invaded untreated MDA-MB-231 cells (**C**), DMSO-pretreated cells with GA (**D**), and PF-4708671-pretreated cells with GA (**E**) using the Boyden chamber system. Scale bar: 100 μm. (**F**) The bar graph shows the number of invaded MDA-MB-231 cells and DMSO- and PF-4708671-pretreated cells with or without GA, compared to the untreated cells, the control. (**G**) Representative Western blots showing the impact of the p70S6K1 blockade using PF-4708671 on GA-induced enolase-2, cathepsine D, CapG, and galectin-3, compared with the negative control, DMSO-pretreated MDA-MB-231 cells, and the control, the untreated cells. The results are presented as the mean ± SD of three independent experiments. (*), (**), (***), and (****) signify a statistically significant difference (*p* < 0.05, *p* < 0.01, *p* < 0.001, and *p* < 0.0001) compared with the control.

For therapeutic purposes, the administration of a specific pharmacological inhibitor of p70S6K1 such as PF-4708671 would be more applicable in clinical practice than the downregulation of p70S6K1 via siRNA technology. A blockade of p70S6K1 activity was confirmed by the inhibition of the phosphorylation of its substrate, ribosomal protein S6, compared to the basal expression level detected in untreated cells and DMSO-treated cells, the negative control (Figure 5A). A complete loss of GA stimulatory effects on the phosphorylation of the ribosomal protein S6 and ERK1/2 was observed after a blockade of p70S6K1 activity, which was greater on pS6 phosphorylation (100% inhibition) than on ERK1/2 phosphorylation (50% inhibition), revealing the specificity of the p70S6K1 blockade by PF-4708671 (Figure 5B). Next, the impact of PF-4708671 was investigated on GA-induced cell invasion and the expression of selected cancer-related proteins in MDA-MB-231 cells. Regarding cell invasion, the addition of GA to un-pretreated cells and DMSO-pretreated cells (Figure 5D) resulted in a significant increase (1.98-fold, *p* = 0.0015 and 2.12-fold, *p* = 0.0037, respectively, Figure 5F) in the number of invaded cells, compared to the untreated cells, the control (Figure 5C,F). Cell pretreatment with PF-4708671 suppressed GA-induced cell invasion (Figure 5E,F). Additionally, the impact of PF-4708671 administration on the expression level of cancer-related proteins was assessed by Western blot technology. The results showed that GA significantly increased the expression level of selected cancer-related proteins, including enolase-2 (1.25-fold, *p* = 0.00043), CapG (1.3-fold, *p* = 0.045), galectin-3 (1.28-fold, *p* = 0.00004), and cathepsin D (1.68-fold, *p* = 0.00049), compared to the untreated cells, the control (Figure 5G). Interestingly, PF-4708671 pretreatment led to the significant inhibition of GA-induced enolase-2 (0.7-fold, *p* = 0.0058), CapG (0.6-fold, *p* = 0.0022), and galectin-3 (0.6-fold, *p* = 0.040), compared to GA-treated cells. Of note, GA-induced cathepsin D expression levels tended to decrease after PF-4708671 pretreatment (Figure 5G).

### 3.6. Prognostic Values of GA-Induced Cancer-Related Proteins on OS and DFS of BC Patients

**Figure 6:**
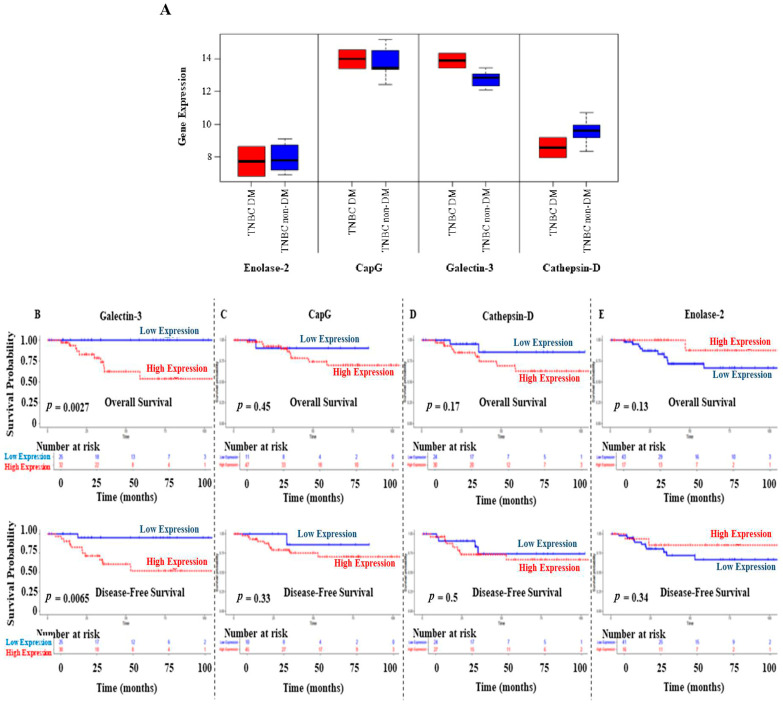
(**A**) **Upregulation of galectin-3 and CapG gene expression detected in DM patients diagnosed with TNBC, while lower expression of enolase-2 and cathepsin D was observed in DM patients, compared to their non-diabetic counterparts**. Data were obtained from the study of Tamez-Peña et al. [34]. The middle line of the boxplots represents the median value; the error bars represent the maximum and minimum values. (**B**–**E**) **Overall and disease-free survival results for galectin-3, CapG, cathepsin D, and enolase-2 in TNBC patients**. Data were obtained from the TCGA. The expression level of each gene was categorized as low or high according to the median value.

Further in silico analyses were performed to validate the expression of enolase-2, CapG, galectin-3, and cathepsin D in DM and non-DM BC patients. Using data from the study conducted by Tamez-Peña et al. [34], the analysis shows increased gene expression levels of CapG and galectin-3 in the DM TNBC patients compared to their non-diabetic counterparts (Figure 6A). In contrast, enolase-2 and cathepsin D showed a lower expression profile in DM TNBC patients. Due to the small sample size, pairwise statistical analysis was insignificant for the comparison between DM and non-DM groups.

Survival analysis was performed on TNBC patients (*n* = 62) from the TCGA data [30]. Interestingly, TNBC patients with high galectin-3 expression levels showed significantly poor OS (*p* = 0.0027) and disease-free survival (DFS) (*p* = 0.0065) compared to those with low galectin-3 expression levels (Figure 6B). Survival results showed a similar pattern in CapG and cathepsin D expression levels despite the insignificant results (Figure 6C,D). In contrast, TNBC patients with high enolase-2 expression levels had favorable OS and DFS, although the analysis was insignificant (Figure 6E).

## 4. Discussion

In search of developing therapeutic strategies against highly invasive BC in T2DM patients that were associated with high gene expression levels of the ribosomal p70S6K1, we investigated the biological impact of p70S6K1 downregulation and the blockade of its kinase activity on the oncogenic effects of GA on TNBC progression. Therefore, p70S6K1 gene expression was downregulated using siRNA transfection technology, and p70S6K1 activity was blocked using a specific pharmacological inhibitor named PF-4708671. The downregulation of p70S6K1 using siRNA technology or the blockade of p70S6K1 activity using PF-4708671 resulted in the suppression of the oncogenic effects of GA, as revealed by increased cell invasion, the overphosphorylation of ribosomal protein S6, and ERK1/2 in TNBC MDA-MB-231 cells. To gain an overview of cancer-related proteins involved in invasive BC development and progression, oncology-related protein arrays were analyzed and revealed the upregulation of capping protein CapG, galectin-3, enolase-2, cathepsin D, and the EGF receptor in GA-treated MDA-MB-231 cells. Mainly involved in cancer progression, the expression of all of these aforementioned proteins was decreased after p70S6K1 downregulation/blockade even in the presence of GA, suggesting p70S6K1 activation as a potential therapeutic target for the fight against BC progression in T2DM patients.

Here, using bioinformatics and the public data repository, the impact of high p70S6K1 expression levels led to a poor prognosis for TNBC patients, while low p70S6K1 expression levels resulted in an increased OS rate of the TNBC patients, revealing the beneficial effect of targeting p70S6K1. In addition, using transcriptomic analysis, p70S6K1 was found to be predominantly expressed in TNBC tissues. In previous studies, the expression level of phospho-p70S6K1, the activated form of ribosomal protein S6 kinase 1, was concomitantly increased in the highly invasive TNBC cell line MDA-MB-231 cultured in the presence of GA [9]. Moreover, phospho-p70S6K1 was reported to be highly expressed in IDC tissues extracted from T2DM patients, while phospho-p70S6K1 was weakly expressed in their non-DM counterpart IDC tissues [14]. No changes in the total form of p70S6K1 expression levels in these IDC tissues were observed between T2DM and their non-DM counterparts [14]. These findings suggest the key role of phospho-p70S6K1 overexpression in TNBC progression under T2DM conditions, which was further explored in vitro by targeting p70S6K1 using siRNA transfection technology and the specific pharmacological inhibitor.

Well known as a cytoplasmic target of Ras/MAPK/mTOR and PI3K/Akt/mTOR pathways, the subsequent phosphorylation of p70S6K1 leads to protein and lipid synthesis in ribosomes, ribosome protein translation, the regulation of cell proliferation, cell cycle progression, DNA damage checkpoints, and telomere length maintenance, as well as cancer cell metastasis, invasion, and chemotherapeutic drug resistance [36,37]. While the downregulation of p70S6K1 using siRNA technology was verified using Western blot analysis based on the detection of its protein, the oncogenic effects of GA were confirmed by the overphosphorylation of ERK1/2 and p70S6K1, as reported in previous studies [9,35,38]. The use of the specific pharmacological inhibitor PF-4708671 resulted in the blockade of p70S6K1 activity, revealed by the inhibition of ribosomal protein S6 phosphorylation. Unlike high therapeutic concentrations of metformin, PF-4708671 was reported to exhibit beneficial effects in vitro and in vivo at lower concentrations, including decreasing basal hepatic glucose production and preventing chronic hyperglycemia effects on oxidative metabolism, suggesting PF-4708671 as a potential anti-diabetic therapeutic agent [39,40].

The stimulation of the cell motility process of the highly invasive TNBC cell line MDA-MB-231 by the addition of GA confirmed our previous study using scratching assay and Matrigel^®^-coated transwell inserts, reported to be associated with enhanced matrix metalloproteinase (MMP)9 activity [9]. While GA stimulated BC cell functions, p70S6K1 knockdown and the blockade of its kinase activity abolished GA oncogenic effects, confirming the key role of p70S6K1 in tumor metastasis [41]. In addition to that, p70S6K1 downregulation/blockade suppressed GA-induced protein S6 and ERK1/2 phosphorylation, the last steps of signaling pathways, resulting in the stimulation of gene expression involved in cancer development and progression [42].

To pinpoint the potential cancer-related proteins involved in the p70S6K1-dependent mechanism of GA-induced BC progression, an oncology array was performed in GA-treated TNBC cells after p70S6K1 knockdown. Various oncogenic protein expressions were upregulated by the addition of GA, while their expressions were decreased after p70S6K1 knockdown even in the presence of GA. The suppression of GA-upregulated cancer-related protein expression after p70S6K1 downregulation confirmed the key role of p70S6K1 in protein synthesis [36]. These oncogenic proteins were gelsolin-like actin-capping protein CapG, galectin-3 (also known as AGER3), enolase 2 (ENO2), cathepsin D (CTSD), and the EGF receptor (EGFR). Capping protein (CapG, also known as macrophage-capping protein, MCP) is a cytoskeletal protein that, when bound to phosphatidylinositol 4,5-biphosphate (PIP_2_), regulates cell motility and invasion through actin polymerization. The latter process was described to be generated by PIPKIγ90 (phosphatidylinositol 4-phosphate 5-kinase type Iγ), whose activation occurred after p70S6K1-mediated phosphorylation [43]. Located in the nucleus and in the cytoplasm, CapG has also been identified as an oncogene, and its overexpression has been associated with poor prognosis and metastasis in several cancers including BC, colorectal, and prostate cancer [44,45]. Predominantly expressed in the cytoplasm, galectin-3, a versatile γ-galactoside-binding lectin with a broad spectrum of multi-functions, translocates from the nucleus to the extracellular cell surface, where it modulates cell–cell and cell–matrix interactions. Galectin-3 plays a key role in tumor progression and metastasis through c-MYC upregulation and YAP1/RalA/RalBP, conferring an aggressive phenotype [46,47]. Additionally, galectin-3 has been described for the selective autophagy of endomembrane damage, likely via the AMPK/mTOR pathway [48]. Furthermore, soluble galectin-3 has been described to be highly secreted by breast cancer and TNBC cells and to be associated with chemotherapy resistance and immunosuppression, suggesting galectin-3 as a diagnostic marker and a potential therapeutic target in BC development and progression [49,50]. Recently reported to be overexpressed in pancreatic β-cells, an elevated plasma galectin-3 level was detected in T2DM patients [51,52]. Mainly involved in accelerated aerobic glycolysis supporting the increased cancer cell metabolic needs (Warburg effect), the glycolytic enzyme enolase-2 (also known as γ-enolase neuron-specific enolase) is upregulated in tumors including BC and lymph node metastases, with its major contribution to tumor progression including cell migration and invasion through actin cytoskeleton remodeling [53,54]. A slight increase in plasma enolase-2 concentration has been reported in T2DM patients, and it was concomitantly enhanced in patients with diabetic neuropathy [55]. In conjunction with our in vitro findings, it would be interesting to confirm an enhancement of plasma enolase-2 concentration in T2DM patients diagnosed with highly invasive BC compared to that observed in their non-diabetic counterparts. Cathepsin D is an aspartyl lysosomal protease whose secretion into the tumor microenvironment has been associated with BC metastasis and a poor prognostic marker for BC progression [56]. Recently, using preclinical TNBC models, anti-cathepsin D antibodies were reported to inhibit tumor growth and restore antitumor activity, opening new avenues for TNBC immunotherapy [57]. The development of these anti-cathepsin D antibodies could be of interest for T2DM patients diagnosed with TNBC.

## 5. Conclusions and Future Perspectives

Here, we report the key role of p70S6K1 in GA-stimulated invasive BC progression, which was evaluated after p70S6K1 downregulation using siRNA technology and the p70S6K1 blockade using a specific pharmacological inhibitor PF-4708671. The downregulation of p70S6K1 and the inactivation of its kinase resulted in the suppression of GA oncogenic effects on the highly invasive TNBC cell line MDA-MB-231 functions, as demonstrated by the inhibition of the GA-induced overphosphorylation of the oncogenic signaling protein ERK1/2, GA-stimulated cell invasion, and GA-upregulated cancer-related proteins such as galectin-3, revealed as a potential prognostic marker for DM and invasive BC. Altogether, these findings suggest that phospho-p70S6K1 is a promising therapeutic target and galectin-3 is a potential prognostic biomarker for T2DM patients diagnosed with invasive BC. Further research is needed to explore the potential therapeutic effects of PF-4708671 as anti-diabetic and antitumorigenic agent using in vitro and in vivo models. It is also noteworthy to mention that the in silico analysis using the study of Tamez-Peña and colleagues [34] was performed on two diabetic TNBC patients, a very small sample size to extrapolate any significant or meaningful results. To our knowledge, there are no other publicly available data that can be used for further in silico validation. Thus, further clinical investigation reporting p70S6K1 gene expression levels in T2DM patients diagnosed with TNBC would be of interest in a larger cohort to validate this genetic association.

## Data Availability

The raw data supporting the conclusions of this article will be available by the authors on request.
